# Psychological, disability, and somatosensory characteristics across different risk levels in individuals with low back pain: A cross-sectional study

**DOI:** 10.1016/j.bjpt.2025.101185

**Published:** 2025-02-11

**Authors:** Hester L. den Bandt, Kelly Ickmans, Ronald Buyl, Lynn Leemans, Jo Nijs, Lennard Voogt

**Affiliations:** a Research Centre for Health Care Innovations, University of Applied Sciences Rotterdam, Rotterdam, The Netherlands; b Pain in Motion Research Group, Department of Physical Therapy, Human Physiology and Anatomy, Faculty of Physical Education and Physical Therapy, Vrije Universiteit Brussel, Brussels, Belgium; c Department of Physical Medicine and Physical Therapy, University Hospital Brussels, Brussels, Belgium; d Movement & Nutrition for Health & Performance research group (MOVE), Department of Movement and Sport Sciences, Faculty of Physical Education and Physical Therapy, Vrije Universiteit Brussel, Brussels, Belgium; e Rehabilitation Research Department, Vrije Universiteit Brussel, Brussels, Belgium; f Department of Biostatistics and Medical Informatics, Vrije Universiteit Brussel, Brussels, Belgium; g Department of Health and Rehabilitation, Unit of Physical Therapy, Institute of Neuroscience and Physiology, Sahlgrenska Academy, University of Gothenburg, Sweden. University of Gothenburg, Sweden

**Keywords:** Non-specific low back pain, Prognostic indicators, Psychosocial, Sensitivity, Subgroups

## Abstract

**Background:**

Current low back pain (LBP) treatment might be improved by tailoring treatments to subgroup characteristics. The Start Back screening Tool (SBT) and Central Sensitization Inventory (CSI) classify people with LBP into subgroups. It is currently unknown whether linear trends exist regarding somatosensory changes, psychological characteristics, and physical disability across severity levels of the SBT and CSI in patients with LBP.

**Objective:**

To investigate whether linear trends in psychological variables (kinesiophobia, pain catastrophizing), disability, and somatosensory characteristics exist in a sample of people with acute and chronic LBP in primary care across severity levels based on the SBT and CSI.

**Methods:**

Participants with LBP were recruited in primary care. Demographic, psychological, and disability data were obtained. A comprehensive Quantitative Sensory Testing (QST) protocol was followed. Linear contrast analysis was conducted.

**Results:**

Kinesiophobia and pain catastrophizing show significant positive linear trends across the subgroups based on the SBT (*p* < 0.001) and CSI (*p* < 0.001 to *p* = 0.01). Heat pain threshold at the lower leg (*p* = 0.005) and pressure pain threshold at the lumbar region and lower leg (*p* = 0.02 and *p* = 0.04, respectively) show significant negative linear trends within the SBT. Negative linear trends in sensory changes exist within CSI for all pressure pain thresholds and a positive linear trend was seen in δ conditioned pain modulation at the thumb (*p* = 0.03).

**Conclusion:**

Kinesiophobia, pain catastrophizing, disability, and pain intensity are positively related with the severity levels based on the SBT and CSI in our participants. Results of somatosensory changes were sometimes related to the severity levels.

## Highlights

• The extent of psychological factors increases as the SBT/CSI severity level increases.
• Increased somatosensory sensitivity was seen often as the severity level increases.
• The results provide intention for tailoring treatment for the specific LBP subgroups.

## Introduction

Low back pain (LBP) is a complex health problem characterized by many neurophysiological and psychosocial factors that might be involved, like altered sensory processes in the brain, pain catastrophizing, and kinesiophobia.^1^, ^2^, ^3^, ^4^ Marcuzzi et al.^5^ showed that neurophysiological changes in the somatosensory system are present when acute LBP develops into persistent LBP.^5^ They concluded that pain-related psychological variables were significantly lower in those with recovered LBP compared to those with persistent LBP.^5^ Central sensitization (CS) is a neurophysiological process that might be part of the complex LBP biology, indicating that symptoms of CS may be present in a subgroup of people with LBP.^6^^,^^7^ To identify symptoms related to CS the Central Sensitization Inventory (CSI) has been developed, containing questions concerning cognitive, somatic, and emotional health-related symptoms.^8^^,^^9^

People with LBP can be divided into subgroups based on clinical characteristics. By tailoring to these characteristics current treatment might be improved. Stratified care is defined as grouping people with LBP in risk levels to target interventions specific to that risk level.^10^, ^11^, ^12^ This approach has been suggested for LBP-rehabilitation and evidence shows promising outcomes for both cost-effectiveness and patient outcomes.^12^^,^^13^ The Start Back screening Tool (SBT), a commonly used questionnaire in primary care, indicating the odds of unfavorable prognosis, stratifies people with LBP into risk levels.^14^^,^^15^ The SBT contains several pain-related and psychological questions.^15^ Besides the SBT, other measures have been developed to assist clinicians in tailoring treatments, like the CSI. This questionnaire also divides people into subgroups: low, medium, and high severity levels.^9^

Quantitative sensory testing (QST) is used as a proxy for CS in clinical studies, and QST-measurements attempt to objectify sensitivity changes in the somatosensory system.^16^ Based on the findings by Marcuzzi et al., the hypothesis is formulated that changes in the somatosensory system and psychological and disability factors may differ between the risk/severity levels of the SBT and CSI in patients with LBP.^5^ Additionally, it is hypothesized that there is an overlap in identifying such LBP subgroups by using the SBT and CSI.

Stratifying patients fits within the idea of precision medicine, defined as the ability to classify patients into subgroups that differ in their susceptibility, biology, or prognosis of a particular disease or in their response to a specific treatment.^17^ A tentative step towards precision medicine for patients with LBP in primary care is taken with this innovative exploratory study to outline psychological and somatosensory characteristics per subgroups. Studying changes in the psychological factors combined with the alterations in the somatosensory system in these patients is novel. If characteristics are present in these LBP subgroups, this can support clinicians in interpreting SBT- and/or CSI-defined subgroup classification, which in turn might assist in determining appropriate treatment for patients with LBP seen in primary care.

Hence, the aim of this study is to investigate whether a linear trend exists across severity levels of both the SBT and CSI regarding kinesiophobia, pain catastrophizing, pain intensity, disability, and changes in the somatosensory system in people with acute and chronic LBP in primary care. To know whether both questionnaires measure their own construct, the level of agreement is assessed in identifying subgroups between the SBT and CSI in the same population.

## Methods

### Study design and setting

This cross-sectional study is reported according to the Strengthening the Reporting of Observational studies in Epidemiology (STROBE) Statement.^18^ From November 2016 to April 2019, people with LBP, visiting Dutch primary care physical therapy practices, were recruited. Ethical approval was provided by the Ethical committee of Maasstad Hospital, Rotterdam, The Netherlands (T2016–38).

### Participants

Inclusion criteria were: age between 18 and 65 years, having had LBP for at least one week with or without referred pain in one or both lower extremities and having a mean pain intensity reported on the 0–100 mm Visual Analogue Scale (VAS) of ≥ 30 mm during the last week. Exclusion criteria were: pregnancy; LBP after surgery or trauma; psychiatric diagnosis determined by a psychiatrist; people with fibromyalgia, chronic fatigue syndrome, or rheumatoid arthritis; LBP due to referred pain from internal organs; and inability to write or read Dutch.

### Procedure

Participants were screened for eligibility according to the Dutch LBP guideline of the Royal Dutch Society for Physiotherapy by their treating physical therapist and were informed about this study.^19^ Patients received an information leaflet about the objectives and content of the study. The first author (HdB), an experienced physical therapist trained in performing QST-measurements, executed all measurements. HdB was blinded to the medical record and all other study data of the participants. Prior to enrollment, all participants provided written informed consent.

First, participants provided demographic information about sex, age, most painful site of their LBP, and the present type of pain (by self-completing the questionnaire “painDETECT”). The other questionnaires: SBT, CSI, Tampa scale for kinesiophobia, Pain Catastrophizing Scale, Roland Morris Disability Questionnaire, and VAS for pain severity were also self-administered. To avoid test order effect, questionnaires were administered in an alternately structured order: participant A started with questionnaire 1 (and ended with questionnaire 7), participant B started with questionnaire 2 (and ended with questionnaire 1), participant C started with questionnaire 3 (and ended with questionnaire 2) and so on. After completing the questionnaires, the study was continued with the QST-measurements.

### Measurements

The *Start Back screening Tool (SBT)* identifies to what extent patients with LBP seen in primary care are at risk for a poor prognosis. It classifies patients in one of three risk levels (low-, medium-, high-risk).^14^ The SBT consists of nine questions of which eight questions need to be answered with “true/false” and one on a 5-point Likert-scale.^20^ The Dutch version generates sufficient valid and reliable data and shows substantial reproducibility.^20^

The *Central Sensitization Inventory* (CSI) measures CS related symptoms and consists of 25 CS related questions, to be answered on a 5-point Likert-scale (0 = never, 4 = always). The Central Sensitization Inventory Symptom Severity Calculator determines patient related severity level (low, medium, high) of the CSI. The test-retest reliability of the Dutch version is excellent.^21^ The intraclass correlation coefficient (ICC) varies from 0.88 to 0.91.^21^ The sensitivity and specificity of the questionnaire is 81 % and 75 %, respectively.^22^

The *Tampa scale for kinesiophobia (TSK)* assesses kinesiophobia. It consists of 17 statements that need to be scored on a 4-point Likert scale (1 = highly disagree, 4 = highly agree).^23^ A cut-off score of ≥37/68 indicates kinesiophobia.^23^ The Dutch version of the TSK (TSK-DV) shows high internal consistency (Cronbach's alpha range 0.68, 0.80), good construct and criterion validity, and an ICC of >0.7.^24^^,^^25^

The *Pain Catastrophizing Scale (PCS)* measures the degree of pain catastrophizing by assessing three related subscales: magnification, rumination, and helplessness.^26^ It consists of 13 statements that need to be scored on a 5-point Likert scale (0 = not at all, 4 = all the time).^26^ A cut-off score of ≥30/52 is used to establish “pain catastrophizing”.^26^ The PCS shows good test-retest reliability and internal consistency.^27^^,^^28^

The *Roland Morris Disability Questionnaire (RDQ)* assesses the functional status of people with LBP.^29^ This questionnaire contains 24-items in which the answer options are “yes/no”.^30^ Total score is calculated by adding up the number of “yes” answers ranging from 0 (=no disability) to 24 (= maximal disability).^31^ Higher scores indicating a higher level of disability.^31^ The Dutch version of the RDQ is a reliable questionnaire (ICC= 0.91).^29^

The *Visual Analogue Scale (VAS)* measures pain intensity of people with LBP. This self-reported scale has a horizontal line of 100 mm. The score varies from 0 mm (no pain) to 100 mm (unbearable pain).^32^ Participants are requested to mark the line that corresponds most closely with the pain intensity they currently experience.^32^ Despite its low content validity, the VAS is recommended to measure and report pain in clinical trials in people with LBP.^32^^,^^33^ The ICC varies from 0.73 to 0.85.^34^

The *painDETECT* discriminates between nociceptive and neuropathic pain mechanisms.^35^ It consists of seven questions about the quality of neuropathic pain symptoms with a 5-point Likert-scale (0=never, 5=very strongly), one question about radiating pain answered by “yes/no”, and four pictures that describe the pain course pattern. The participant has to mark the picture that describes the pain course best. A total score ≤ 12 is predominantly nociceptive pain and a total score of ≥ 19 is predominantly neuropathic pain.^35^ It is a valid, reliable screening tool and it has been adequately translated into Dutch.^35^^,^^36^ The ICC is 0.91.^37^

### QST-measurements

To determine the testing site for the QST-measurements, participants indicated the most painful side of their LBP. If the pain was the same between sides, the right side was chosen. QST-measurements were performed at the following locations: thumb, lower back (2 cm lateral to the spinous process of L4), and gastrocnemius muscle-tendon junction. During the measurements the participants lied prone and the locations were marked. The measurements started with heat pain threshold (HPT). After a five-minute pause, pressure pain threshold (PPT) was measured. After another five minutes, temporal summation (TS) was assessed. After another five minutes pause, conditioned pain modulation (CPM) was assessed. The whole procedure took one hour.

*Heat Pain Threshold* (HPT) measurements were conducted with an increasing stimulus (1 °C /sec, 32 °C baseline and 50 °C cut-off, 8 cm^2^ thermode), using the TSA 2001-II (MEDOC, Israel). Participants were instructed to say ‘stop’ when the sensation first became uncomfortable.^38^ The measurements were performed twice on each location (i.e., thumb, lower back (2 cm lateral to the L4 spinous process), and the gastrocnemius muscle-tendon junction) with an interval of 30 s. For analysis, the mean scores of each location were calculated and used. Indication for enhanced sensitivity in the somatosensory system is lower HPTs at non-segmental level.^39^ HPT measurements have proved to be of acceptable reliability.^40^

*Pressure Pain Threshold* (PPT) measurements were performed with a handheld pressure algometer (Wagner Instruments, FDX 50 Algometer, Greenwich, USA,). The circular probe has a 1 cm diameter and the measurement was performed by applying an increasing stimulus (1 kg/s). The instruction was to say ‘stop’ when the sensation first became uncomfortable.^38^ PPTs were also assessed twice at the same locations as the HPT with an interval of 30 s and the mean of each location was calculated and used for analysis. Lower remote PPTs at non-segmental levels are also indicative of CS.^39^ PPT measurements show acceptable test-retest reliability.^40^

*Temporal summation* (TS) was measured by applying 10 consecutive identical nociceptive stimuli at the same test locations described above.^41^ Pain sensation will increase if the neuronal output amplifies, which mediates TS.^41^ The stimuli were applied at the previously determined mean PPT intensity. This pressure was maintained for one second before starting the measurement. At a rate of approximately 2 kg/s for each stimulus, the pressure was increased. Stimuli were presented with an interstimulus interval of one second. By means of a verbal numeric rating scale (0=no pain, 10 =unbearable pain) for the pain intensity, the participants had to rate the first, fifth, and tenth stimulus. The result for TS is calculated by the difference between the tenth and the first verbal numeric rating scale score. The reliability is acceptable.^40^

Endogenous pain inhibition is measured by means of the *Conditioned Pain Modulation* (CPM) paradigm^42^ using the Thermo Scientific™ VersaCool™ refrigerated circulating bath (ThermoFisher Scientific, Newington, U.S.A.) and the pressure algometer. The painful conditioning stimulus was done by immersing the participant's hand, contralateral to the most painful side, in a cold water bath of 12 °C. Participants were instructed to keep their hand in the water as long as bearable for a maximum of two minutes. On each test location, PPTs were taken as the test stimulus, once their hand was removed out of the water. In order: 1) thumb, 2) lower back, 3) gastrocnemius muscle-tendon junction. Mean score of each location was calculated and used for analysis. For drawing a conclusion about endogenous pain inhibitory capacity, post-conditioning scores were subtracted from pre-conditioning scores. The reliability is acceptable.^40^

### Analysis and statistics

This study was performed as a secondary analysis of a case-control study which investigated whether differences in QST-measurements exist between people with acute and chronic LBP and healthy controls.^43^ For data analysis IBM SPSS Statistics for Windows Version 25.0 (Armonk, NY: IBM Corp.) was used. Demographic data were presented as mean (standard deviation). The participants were divided into acute (0–12 weeks) and chronic LBP (≥ 12 weeks).^44^ The entire dataset was checked for completeness. Incomplete questionnaires were removed for analysis. Outliers were identified by checking boxplots. Only the outliers who exceeded at least 1.5 times the inter quartile range,^45^ were removed from the data analysis. Cohen's kappa was calculated estimating the agreement in identifying subgroups identified by the SBT and CSI (categorical variables): whether participants belonging to the SBT high risk level, were in the CSI high severity level. The interpretation of Cohen's kappa was according to Landis and Koch^46^ (<0.00 poor, 0.00–0.20 slight, 0.21–0.40 fair, 0.41–0.60 moderate, 0.61–0.80 substantial, 0.81–1.00 almost perfect). Spearman's correlation calculation was done between the CSI and painDETECT to determine if participants with positive painDETECT were a possible confounder. The interpretation was according to Schober et al.^47^ (0.00–0.10 negligible, 0.10–0.39 weak, 0.40–0.69 moderate, 0.70–0.89 strong and 0.90–1.00 very strong). A linear contrast analysis was performed. Conditionally, group mean of the three risk/severity levels of the SBT and CSI need to differ significantly. Therefore, one-way ANOVA was applied, based on the significance of the Levene's test (*p* < 0.01). If it met this requirement linear contrast analysis was performed. Dependent variables were: QST measurements and questionnaires (except PainDETECT). Independent variables were: risk/severity levels of SBT or CSI and participants divided into the acute and chronic LBP group. Adjustments were made for sex and age.

## Results

### Participants and descriptive data

One hundred participants with LBP with a mean age of 42.36 (SD 10.84) years participated. Forty-four participants were male and 56 were female. Forty-seven participants had acute and 53 had chronic LBP (Table 1). On the painDETECT, 57.1 % of the participants scored negative (nociceptive pain), 23.5 % scored ambiguous (unclear pain mechanism), and 19.4 % of participants scored positive (neuropathic pain). The CSI scores correlated moderately with the painDETECT scores (*r* = 0.47; *p* < 0.001).^47^ During recruitment two participants reported that this study was too time consuming and therefore discontinued study participation. One participant appeared to be insufficiently proficient in the Dutch language and was excluded. Two more participants were excluded: one suffering from fibromyalgia and one having had low back surgery. The recruitment ended on reaching the required 100 participants. Four SBT, one CSI, and one painDETECT questionnaires were excluded from further analysis due to incompleteness. Due to technical problems, the HPT measurements consisted of 91 data points instead of 100.

**Table 1:** Descriptive statistics of the study sample of patients with low back pain in primary care.

	Acute LBP, N = 47	Chronic LBP, N = 53	
Age (mean/SD)	43.7 (11.02)	41.17 (10.64)	
Sex	Women, *n* = 22	Women, *n* = 34	
	Men, *n* = 25	Men, *n* = 19	
TSK (mean/SD)	32.31 (6.58)	34.78 (7.99)	
PCS (mean/SD)	15.23 (8.42)	21.85 (12.81)	
VAS_mean_ (mean/SD)	37.06 (21.1)	55.77 (22.91)	
RDQ (mean/SD)	7.40 (4.81)	10.53 (5.18)	
SBT	Low risk level, *n* = 29 (61.7 %)	Low risk level, *n* = 20 (37.7 %)	N = 4 missing data
	Medium risk level, *n* = 16 (34.0 %)	Medium risk level, *n* = 21 (39.6 %)	
	High risk level, *n* = 1 (2.2 %)	High risk level, *n* = 9 (17.0 %)	
			
CSI	Low sev. level, *n* = 12 (25.5 %)	Low sev. level, *n* = 4 (7.5 %)	N = 1 missing data
	Medium sev. level, *n* = 26 (55.3 %)	Medium sev. level, *n* = 27 (50.9 %)	
	High sev. level, *n* = 9 (19.1 %)	High sev. level, *n* = 21 (39.6 %)	
PainDETECT	Nociceptive, *n* = 32 (68.1 %)	Nociceptive, *n* = 25 (48.1 %)	N = 1 missing data
	Ambiguous, *n* = 11 (23.4 %)	Ambiguous, *n* = 12 (23.1 %)	
	Neuropathic, *n* = 4 (8.5 %)	Neuropathic, *n* = 15 (28.8 %)	

CSI, Central Sensitization Inventory; LBP, Low Back Pain; PCS, Pain Catastrophizing Scale; RDQ, Roland Morris Disability Questionnaire; SBT, Start Back screening Tool; SD, standard deviation; TSK, Tampa Scale for Kinesiophobia; VAS, Visual Analogue Scale

### Level of agreement between SBT and CSI

The level of agreement (95 % CI) between the SBT and CSI was 0.10 (−0.03, 0.23), which indicates no agreement.^46^

### Linear trends of the start back screening tool with psychosocial and disability questionnaires and QST-measurements

All questionnaires showed positive significant linear trends across the risk levels (TSK, PCS, VAS_mean_, RDQ, all *p* < 0.001) with adjustments for sex and age. Both HPT_thumb_ and HPT_gastroc_ showed negative significant linear trends across the risk levels (HPT_thumb_, *p* = 0.03; HPT_gastroc_, *p* = 0.005). Both PPT_L4_ and PPT_gastroc_ showed negative significant linear trends across the risk levels (PPT_L4_, *p* = 0.02, PPT_gastroc_, *p* = 0.04). None of the TS measurements showed significant linear trends across the risk levels. Only the δCPM_thumb_ showed a positive significant linear trend across the risk levels (δCPM_thumb_, *p* = 0.04) (Table 2).

**.Table 2:** Descriptive statistics of the study sample of patients with low back pain per risk level and results of linear trends based on the Start Back Screening Tool.

Variable	Acute LBP (N = 47)	Results Mean (SD)	Chronic LBP (N = 53)	Results Mean (SD)	Linear contrast sig.	95% CI
TSK^a^						
Low risk	29	30.00 (6.16)	20	31.20 (6.74)	*p* < 0.001	(3.00, 9.39)
Medium risk	14	36.64 (5.29)	19	36.37 (8.14)		
High risk	1	40.00	9	40.44 (6.52)		
PCS^b^						
Low risk	29	11.17 (6.30)	19	11.95 (8.04)	*P* < 0.001	(11.30, 20.06)
Medium risk	16	21.63 (7.29)	21	23.90 (12.13)		
High risk	1	30.00	9	35.89 (7.08)		
VAS_mean_						
Low risk	29	27.90 (15.58)	20	40.70 (21.00)	*P* < 0.001	(10.94, 30.32)
Medium risk	16	54.06 (19.39)	21	64.14 (21.75)		
High risk	1	56.00	9	67.78 (13.16)		
RDQ						
Low risk	29	5.18 (3.22)	20	6.65 (3.54)	*P* < 0.001	(2.87, 7.09)
Medium risk	16	11.00 (4.65)	21	12.62 (4.99)		
High risk	1	17.00	9	12.78 (4.24)		
HPT thumb^c^ (֯C)						
Low risk	26	45.74 (3.40)	18	45.66 (2.94)	*P* = 0.03	(−3.41, −0.10)
Medium risk	15	45.85 (2.66)	20	45.18 (2.74)		
High risk	1	38.86	8	44.29 (4.92)		
HPT L4^c^ (֯C)						
Low risk	26	45.25 (3.44)	18	44.85 (2.59)	*P* = 0.05	(−3.09, 0.00)
Medium risk	15	45.31 (2.71)	20	44.56 (2.93)		
High risk	1	40.92	8	43.38 (3.45)		
HPT gastroc^c^ (֯C)						
Low risk	26	46.24 (2.23)	18	46.38 (1.67)	*P* = 0.005	(−2.78, −0.49)
Medium risk	15	46.53 (2.09)	20	46.00 (2.79)		
High risk	1	42.67	8	43.90 (4.13)		
PPT thumb (kg/cm^2^)						
Low risk	29	8.58 (3.81)	20	7.93 (3.73)	*P* = 0.15	(−2.76, 0.43)
Medium risk	16	8.54 (3.17)	21	6.97 (2.63)		
High risk	1	7.79	9	6.30 (2.97)		
PPT L4 (kg/cm^2^)						
Low risk	29	9.69 (5.50)	20	7.90 (4.05)	*P* = 0.02	(−4.29, −0.29)
Medium risk	16	7.16 (4.29)	21	5.82 (2.92)		
High risk	1	10.81	9	4.56 (2.29)		
PPT gastroc (kg/cm^2^)						
Low risk	29	7.77 (3.77)	20	6.74 (3.19)	*P* = 0.04	(−2.68, −0.04)
Medium risk	16	7.32 (2.80)	21	5.58 (1.85)		
High risk	1	5.27	9	5.21 (1.70)		
TS thumb (NRS)						
Low risk	29	1.97 (2.08)	20	2.25 (2.57)	*P* = 0.61	(−0.91, 1.52)
Medium risk	16	1.94 (2.72)	21	2.29 (2.81)		
High risk	1	4.00	9	2.00 (1.58)		
TS L4 (NRS)						
Low risk	29	2.24 (1.90)	20	2.60 (2.46)	*P* = 0.66	(−1.32, 0.85)
Medium risk	16	2.13 (2.25)	21	2.33 (2.06)		
High risk	1	2.00	9	1.89 (1.76)		
TS gastroc (NRS)						
Low risk	29	2.10 (2.19)	20	2.65 (1.93)	*P* = 0.74	(−0.89, 1.24)
Medium risk	16	1.38 (2.55)	21	2.10 (1.84)		
High risk	1	4.00	9	2.33 (1.58)		
δCPM thumb (kg/cm^2^)						
Low risk	29	−2.28 (1.88)	20	−2.49 (1.59)	*P* = 0.04	(0.04, 1.92)
Medium risk	16	−1.92 (2.38)	21	−1.59 (1.57)		
High risk	1	−2.26	9	−1.08 (1.35)		
δCPM L4 (kg/cm^2^)						
Low risk	29	−3.11 (1.85)	20	−3.27 (2.12)	*P* = 0.16	(−0.28, 1.72)
Medium risk	16	−3.11 (1.74)	21	−3.27 (2.07)		
High risk	1	−3.97	9	−1.93 (1.61)		
δCPM gastroc (kg/cm^2^)						
Low risk	29	−2.19 (1.47)	20	−2.05 (1.74)	*P* = 0.09	(−0.10, 1.33)
Medium risk	16	−2.04 (1.24)	21	−1.79 (1.17)		
High risk	1	−1.21	9	−1.44 (1.41)		

CI, confidence interval; CPM, Conditioned Pain Modulation; HPT, Heat Pain Threshold; LBP, low back pain; Low leg, lower leg; NRS, Numeric Rating Scale; PCS, Pain Catastrophizing Scale; PPT, Pressure Pain Threshold; RDQ, Roland Morris Disability Questionnaire; SD, Standard Deviation; Sig, significance; TS, Temporal Summation; TSK, Tampa Scale for Kinesiophobia; VAS, Visual Analogue Scale.

a *n* = 96.

b *n* = 99.

c *n* = 9 missing.

### Linear trends of the central sensitization inventory with psychosocial and disability questionnaires and QST-measurements

All questionnaires showed positive significant linear trends across the severity levels (TSK and PCS, *p* < 0.001; VAS_mean,_ *p* = 0.01; RDQ, *p* = 0.001) with adjustments for sex and age. All PPT measurements showed negative significant linear trends across the severity levels (PPT_thumb_, *p* = 0.009, PPT_L4_, *p* = 0.012, PPT_gastroc_, *p* = 0.001). The δCPM_thumb_ showed a positive significant linear trend across the severity levels (*p* = 0.03) (Table 3).

**Table 3:** Descriptive statistics of the study sample of patients with low back pain per severity level and results of linear trends based on the Central Sensitization Inventory.

Variable	Acute LBP (N = 47)	Results Mean (SD)	Chronic LBP (N = 53)	Results Mean (SD)	Linear contrast sig.	95% CI
TSK^a^						
Low sev. Level	11	31.55 (5.91)	4	28.75 (3.77)	*P* < 0.001	(3.52, 10.28)
Medium sev. Level	25	30.92 (5.91)	26	33.46 (8.20)		
High sev. Level	9	37.11 (7.56)	20	37.35 (7.58)		
PCS^b^						
Low sev. Level	12	12.67 (7.43)	4	5.50 (2.89)	*P* < 0.001	(5.07, 15.45)
Medium sev. Level	26	14.19 (7.82)	26	21.50 (11.86)		
High sev. Level	9	21.67 (8.96)	21	24.71 (13.01)		
VAS_mean_						
Low sev. Level	12	32.58 (17.52)	4	35.75 (20.79)	*P* = 0.01	(3.05, 24.90)
Medium sev. Level	26	34.12 (21.27)	27	55.56 (23.71)		
High sev. Level	9	51.56 (20.70)	21	59.67 (21.71)		
RDQ						
Low sev. Level	12	5.33 (4.05)	4	5.50 (3.70)	*P* = 0.001	(1.61, 6.55)
Medium sev. Level	26	7.15 (4.43)	27	10.22 (4.66)		
High sev. Level	9	10.89 (5.35)	21	11.90 (5.67)		
HPT thumb^c^ (֯C)						
Low sev. Level	12	46.54 (1.78)	4	47.21 (2.48)	*P* = 0.28	(−2.53, 0.75)
Medium sev. Level	23	45.21 (3.52)	24	44.70 (3.27)		
High sev. Level	8	44.97 (4.18)	19	45.52 (3.34)		
HPT L4^c^ (֯C)						
Low sev. Level	12	46.63 (1.97)	4	45.06 (1.73)	*P* = 0.24	(−2.39, 0.62)
Medium sev. Level	23	44.56 (3.03)	24	44.80 (3.03)		
High sev. Level	8	44.61 (4.42)	19	43.96 (2.84)		
HPT gastroc (֯C)						
Low sev. Level	12	47.44 (1.43)	4	46.42 (1.24)	*P* = 0.43	(−1.58, 0.68)
Medium sev. Level	23	45.55 (2.15)	24	45.98 (2.42)		
High sev. Level	8	46.25 (2.70)	19	45.67 (3.46)		
PPT thumb (kg/cm^2^)						
Low sev. Level	12	10.25 (2.87)	4	9.81 (4.45)	*P* = 0.009	(−3.60, −0.52)
Medium sev. Level	26	8.43 (3.38)	27	7.18 (3.18)		
High sev. Level	9	6.53 (3.74)	21	6.61 (2.81)		
PPT L4 (kg/cm^2^)						
Low sev. Level	12	11.23 (4.19)	4	10.09 (6.33)	*P* = 0.01	(−4.78, −0.60)
Medium sev. Level	26	8.49 (5.32)	27	6.76 (4.34)		
High sev. Level	9	6.13 (4.65)	21	5.84 (2.57)		
PPT gastroc (kg/cm^2^)						
Low sev. Level	12	9.49 (2.77)	4	9.29 (3.67)	*P* = 0.001	(−3.48, −0.91)
Medium sev. Level	26	7.28 (3.47)	27	6.06 (2.75)		
High sev. Level	9	5.76 (2.76)	21	5.39 (1.66)		
TS thumb (NRS)						
Low sev. Level	12	2.00 (2.22)	4	0.75 (0.96)	*P* = 0.57	(−0.87, 1.55)
Medium sev. Level	26	2.19 (2.25)	27	2.15 (2.92)		
High sev. Level	9	1.67 (2.69)	21	2.67 (1.98)		
TS L4 (NRS)						
Low sev. Level	12	2.42 (2.35)	4	1.00 (2.58)	*P* = 0.55	(−0.78, 1.45)
Medium sev. Level	26	2.08 (1.94)	27	2.74 (2.57)		
High sev. Level	9	2.78 (2.28)	21	2.19 (1.83)		
TS gastroc (NRS)						
Low sev. Level	12	2.00 (2.45)	4	1.50 (1.29)	*P* = 0.69	(−1.26, 0.85)
Medium sev. Level	26	2.08 (2.04)	27	2.59 (1.91)		
High sev. Level	9	1.44 (3.05)	21	2.29 (1.79)		
δCPM thumb (kg/cm^2^)						
Low sev. Level	12	−2.46 (1.36)	4	−3.44 (0.75)	*P* = 0.03	(0.07, 1.95)
Medium sev. Level	26	−2.30 (2.16)	27	−1.97 (1.80)		
High sev. Level	9	−1.14 (2.22)	21	−1.64 (1.57)		
δCPM L4 (kg/cm^2^)						
Low sev. Level	12	−3.48 (1.36)	4	−2.54 (1.98)	*P* = 0.74	(−1.17, 0.84)
Medium sev. Level	26	−2.74 (1.93)	27	−3.10 (1.92)		
High sev. Level	9	−3.42 (1.99)	21	−2.97 (2.24)		
δCPM gastroc (kg/cm^2^)						
Low sev. Level	12	−2.72 (1.61)	4	−2.92 (2.69)	*P* = 0.08	(−0.09, 1.36)
Medium sev. Level	26	−1.82 (1.37)	27	−1.88 (1.44)		
High sev. Level	9	−1.86 (1.10)	21	−1.67 (1.16)		

CI, confidence interval; CPM, Conditioned Pain Modulation; HPT, Heat Pain Threshold; LBP, low back pain; Low leg, lower leg; NRS, Numeric Rating Scale; PCS, Pain Catastrophizing Scale; PPT, Pressure Pain Threshold; RDQ, Roland Morris Disability Questionnaire; SD, Standard Deviation; sev, severity; Sig, significance; TS, Temporal Summation; TSK, Tampa Scale for Kinesiophobia; VAS, Visual Analogue Scale.

a *n*=96.

b *n*=99.

c *n*=9 missing.

## Discussion

This study investigated linear trends in psychological factors, pain-related disability, and somatosensory sensitivity across risk/severity levels of the SBT and CSI in primary care patients with acute and chronic LBP. All questionnaire data (TSK, PCS, VAS_mean,_ and RDQ) showed positive linear trends across the risk/severity levels of the SBT and CSI. This indicates that if the risk/severity levels increase the degree of psychological factors, pain related disability, and pain intensity also increases. Regarding the QST-measurements, HPT_thumb_, HPT_gastroc_, PPT_L4,_ and PPT_gastroc_ showed negative linear trends across the risk levels of the SBT. This indicates that when the risk levels increase, these HPTs and PPTs become more sensitive in our sample. The δCPM_thumb_ showed positive linear trends across the risk levels of the SBT. This indicates that the endogenous inhibitory system functions more poorly when the risk levels increase. Concerning the severity levels of the CSI, all PPT measurements showed negative, and δCPM_thumb_ showed positive linear trends. This shows that as the severity level increases, the PPTs decrease, and that the endogenous inhibitory system is working less efficiently. None of the TS measurements, HPT_L4_, δCPM_L4_ or δCPM_gastroc,_ showed a significant linear trend across the risk/severity levels of the SBT and CSI. The results showed a slight level of agreement between the SBT and CSI total scores in our participants (Cohen's kappa=0.10).^46^ This means that both questionnaires measure their own construct. It is recommended to use both questionnaires for their own purposes. However, the CSI is supposed to measure CS-related symptoms, but the content validity is unknown.^48^

In this study several QST measurements were used. PPTs measure pain sensitivity in deeper tissue. HPTs measure pain sensitivity in the skin.^49^^,^^50^ Additionally static QST measures, the dynamic QST measures used were TS measurements, testing pain facilitation, and CPM measurements, testing descending inhibitory control.^50^ Due to the differences, the results should be interpreted separately. It was expected that if a static QST test could indicate enhanced sensitivity, a dynamic test would also indicate the same. The reality turned out to be different. All TS measurements showed no significant results and some CPM measurements showed significant differences. Presumably, undergoing a TS measurement requires more concentration and cognitive skills from the person compared to the CPM. Not everyone is equally skilled at this. The CPM data showed mixed results. The CPM effect duration in patients with LBP varied from 0 to 10 mins.^51^, ^52^, ^53^ These measurements took place within 10 min, but the effect duration could have influenced the results. To determine sensitivity changes in the somatosensory system QST is used as a proxy of the underlying mechanism.^16^

Several mean scores of patients with chronic LBP in low and medium risk level (SBT) and medium severity level (CSI) for all questionnaires are higher comparing the means of those in acute LBP for the same risk levels, indicating poorer outcome for these factors. The differences in the mean scores of patients with chronic LBP in low and medium risk level (SBT) and in medium severity level (CSI) for the various QST measurements compared to those with acute LBP for the risk levels indicating more sensitivity (.Table 2, Table 3). These findings between people with acute and chronic LBP are consistent with findings from Glare et al.^54^ and Marcuzzi et al.^5^

A tentative step was made to outline characteristics in the SBT- and CSI-defined subgroups of the population of primary care patients with LBP, with the idea of moving towards tailor-made treatments. The knowledge of these results may have implications for clinical practice: this can vary from promoting self-management with some education for patients with a low risk level, to a more comprehensive behavioral approach such as cognitive behavioral therapy combined with e.g. relaxation training, biofeedback, and pain science education to desensitize the central nervous system in the high risk level.^55^

The strength of this study is its innovative character and the representativeness of the included participants: investigating linear trends across the various risk/severity levels of the SBT and CSI for psychosocial, disability, and QST-variables in primary care patients with LBP.

A limitation of the study is the difference in numbers of participants per subgroup. Despite homogeneity of variance, the results should be interpreted with caution. A limitation of a cross-sectional study is that it is only about an association. Causality cannot be inferred. Lack of information caused some limitation; better interpretation of results can be achieved if more demographic potential confounders like income, race, body mass index, physical condition, and educational level were included.

## Conclusion

This cross-sectional study revealed positive linear trends in psychological and disability factors across risk/severity levels of the SBT and CSI in primary care patients with LBP. It implies that psychological and disability factors are more present. Mainly, PPT measurements revealed negative linear trends across risk/severity levels of the SBT and CSI in our participants. There appears to be an increased sensitivity in the somatosensory system in the high subgroups. The remaining QST measurements revealed varying linear trends to no linear trends across risk/severity levels of the SBT and CSI in our participants. The results hold potential for tailoring treatment for the specific LBP subgroups, although these results require prospective validation in different setting.
